# Sociocultural Implications in the Development of Early Maladaptive Schemas in Adolescents Belonging to Sexual and Gender Minorities

**DOI:** 10.3390/ijerph21080971

**Published:** 2024-07-25

**Authors:** Bruno Luiz Avelino Cardoso, Ana Flávia Azevedo Lima, Fabiana Rachel Martins Costa, Christof Loose, Xi Liu, Matteo Angelo Fabris

**Affiliations:** 1 Laboratory of Studies and Interventions in Cognitive and Contextual Therapies, Department of Psychology, Federal University of Minas Gerais, Belo Horizonte 31270-901, Brazilfabianarachel23@ufmg.br (F.R.M.C.); 2 Institute of Schema Therapy in Cologne (IST-K), 50667 Cologne, Germany; 3 The SchemX Collective, Sydney 2042, Australia; xi@lifetherapy.org; 4 Department of Psychology, University of Turin, 10124 Torino, Italy

**Keywords:** schema therapy, sexual and gender minority, adolescents, LGBTQIA+, psychotherapy, minority stress theory

## Abstract

Culture is a central theme across various theories and disciplines, influencing behavior and self-perception through interactions within social groups, families, and legal systems. This influence extends to the general population and particularly impacts sexual and gender minorities (SGMs), resulting in minority stress that contributes to mental health issues and the development of Early Maladaptive Schemas (EMSs). Adolescents within these groups face typical developmental stressors—such as hormonal changes and societal pressures—compounded by prejudice, increasing their vulnerability to depression, anxiety, stress, substance abuse, and eating disorders. Despite these challenges, Schema Therapy (ST) lacks comprehensive studies on the sociocultural aspects influencing EMS acquisition in SGM adolescents. This theoretical review aims to fill this gap by exploring the impact of society and culture on EMS development within SGM adolescents. We recognize the broad spectrum of cultural influences and emphasize the importance of cultural sensitivity and diversity. This review specifically addresses how societal and cultural dynamics impact SGM individuals, acknowledging that while ethnic or other cultural factors are not the focus of this paper, they merit future research. This manuscript will discuss central topics and their impact on LGBTQIA+ youth, including (1) the background (definition of culture, lack of studies on ST focusing on culture, and studies on adverse psychological outcomes), (2) minority stress theory and prejudice against sexual and gender diversity (distal and proximal stressors and sociocultural aspects), (3) EMSs and unmet emotional needs, (4) ST affirmative strategies (working with schema modes, imagery rescripting, chair work, and photo techniques), and (5) final considerations (limitations and research agenda).

## 1. Introduction

In psychology, several theoretical perspectives are dedicated to the study of culture. In general, these models converge on understanding that culture is a set of habits and practices within a group, community, or society [1,2,3]. However, despite the clinical consensus regarding societal and cultural influence on individuals’ mental health [4,5], some psychological models, such as Schema Therapy (ST), still under-explore this topic. This review aims to identify key factors affecting the mental health of people belonging to sexual and gender minorities (SGM) and to propose affirmative therapeutic strategies using ST for this population.

The need for academic focus in this area is highlighted by Pilkington et al. [6], who emphasized investigating contextual factors involved in the development of Early Maladaptive Schemas (EMSs), with culture being identified as a significant factor. Despite this, a gap remains in the theoretical framework of ST, which places little emphasis on the influence of sociocultural aspects in the development of EMSs, as highlighted in the theories of Minority Stress and Affirmative Therapy [6]. Regarding sexual and gender minorities, individuals who face sociocultural oppression due to deviation from cis-heteronormativity (e.g., LGBTQIA+ individuals), cultural factors play a crucial role in their suffering [7]. Furthermore, the historical portrayal of trans individuals in media—including popular films and TV shows—as dangerous, depraved, and disgusting not only impacts public perception but also leaves a lasting effect on self-conceptualization and the development of EMS [8].

The literature consistently indicates that SGM individuals experience higher rates of depression and anxiety [9,10,11], suicidal ideation [12], self-harm [13], and substance abuse [11,14,15] compared to their cis-heterosexual counterparts. During adolescence, these disparities become more pronounced due to inherent stressors and puberty [16].

Hunter et al. [9] explored whether SGM adolescents experience different stress levels compared to their non-SGM peers. They found that SGM adolescents exhibited higher levels of anxiety and depression and lower levels of well-being. Similarly, Fox et al. [10] identified a heightened prevalence of depressive symptoms among SGM adolescents. These mental health outcomes are closely linked to sociocultural variables, such as how different cultural contexts dictate what is considered right or wrong according to gender, social class, ethnic group, sexual orientation and/or gender identity. These variables can perpetuate prejudice against sexual and gender diversity [17].

In discussing the available literature and integrating it with ST concepts, Cardoso et al. [18] proposed understanding a specific schematic mode for minority social groups, particularly prevalent in the SGM population. The inner critic (oppressive sociocultural) mode is characterized by the internalization of prejudice against sexual and gender diversity, comprising schemas, emotional states, and coping strategies that develop in response to the oppressive environment [18]. The authors argue that this mode develops due to the failure to meet basic emotional needs, which are systematically invalidated in SGM individuals. This invalidation often occurs during childhood and adolescence by family, peers, and broader society. 

However, despite ST’s focus on basic emotional needs and the strategies of reparenting and emotional connection [19], studies of sociocultural factors that impact the development of schemas for SGM individuals are still lacking. Studies conducted by professional bodies in both the USA and Australia highlight a dual concern: the under-representation of SGM-identifying therapists and a lack of familiarity with LGBTQIA+ issues for mainstream therapists [20,21]. This gap in understanding can lead to misattunement and microaggressions in clinical settings, which contradict the principles of limited reparenting that form the foundation of ST [22]. Therefore, the objectives of this review are to theoretically (a) discuss aspects of minority stress theory applied to SGM individuals, (b) discuss basic emotional needs and EMSs in SGM adolescents, and (c) suggest affirmative ST intervention strategies for SGM adolescents.

## 2. Minority Stress and Prejudice against Sexual and Gender Diversity

The minority stress theory posits that, in addition to facing common daily stressors experienced by the general population, SGM individuals encounter specific stressors that can lead to psychological harm and distress [23]. These stressors encompass both distal and proximal stressors. Distal stressors include external victimization such as prejudice, discrimination, restrictive policies and legislation, public stigma, exposure to physical and sexual violence, and family rejection. Persistent exposure to these stressors can contribute to the development of proximal stressors, such as the internalization of cultural beliefs and schemas that trigger and exacerbate mental health problems, like expectations of rejection, internalized homophobia, and the concealment of sexual identity [24].

A study by Meyer [23] suggested that the environment can serve as a source of stress, leading to adverse physical and mental health outcomes and hindering the development of coping resources. Conversely, social support plays a crucial role in attenuating the impact of minority stress [25], reducing the risk of depression and other psychological issues [26].

Feinstein et al. [27] conducted a study involving 199 lesbian women and 215 gay men, with an average age of 31 years, which revealed a correlation between depressive symptoms and the internalization of feelings of homonegativity and sensitivity to rejection among individuals reporting less affirming parental experiences. However, no significant association was found between these symptoms and perceived parental attitudes perceived as more welcoming. Affirmative parental attitudes were identified as protective factors for LGB children concerning their own negative feelings and thoughts about sexual orientation [27]. Yet, they do not shield them from the overt discrimination prevalent within the oppressive sociocultural context. The research also highlighted that affirmative parental attitudes regarding sexual orientation are more critical than general support in other areas [27]. Family acceptance serves as a protective factor for adolescents and young adults against depression, substance abuse, and suicidal ideation and behavior, while also promoting self-esteem, social support, and overall better health [28].

A systematic review of the Brazilian literature gathered information from 12 articles, shedding light on the prevalence of homophobia and discrimination within the school environment. These discriminatory behaviors, often subtle, include acts of violence associated with negative representations of non-heterosexual individuals [29]. An additional study conducted in a school environment in Brazil involved 1016 students aged 13 to 21 years [30]. The results revealed that 60% of students reported feeling unsafe at school due to their sexual orientation, while 43% expressed insecurity related to their gender identity or expression. A total of 64% of participants indicated that there were no specific provisions or were unaware of the existence of welcoming policies for LGBTQ+ students in school regulations. Only 8.3% of students reported the presence of provisions related to sexual orientation, identity, or gender expression in school regulations.

A study conducted with American students found similar results [31]. Approximately 50.6% of LGBTQ+ students reported feeling unsafe at school due to their sexual orientation, and 43.2% due to their gender expression. Regarding the existence of school policies for bullying, harassment, or violence, 23.9% of participants reported no policies in their schools. Of those who reported having some kind of policy, only 12% characterized the policy as comprehensive, covering both gender and sexual orientation issues.

In Europe, about 70% of Council of Europe member countries have implemented actions or legislation aimed at the nondiscrimination of LGBTQ+ student communities [32]. Malta, the Netherlands, Norway, and Sweden stand out as countries that have made the most progress in this regard. However, among the 49 member countries, only 22 have policies aimed at ensuring the safety of LGBTQ+ students in schools, and only 54.5% of these policies address both sexual orientation and identity and gender expression. Additionally, only 7 out of 49 member countries recognize the right to gender self-determination for minors [32]

Based on these data, it is possible to understand how minority stress, particularly distal stressors, can play a role in the acquisition of EMSs. According to ST, EMSs originate from the articulation of three factors: (a) unmet emotional needs (as shown in Table 1), (b) environmental experiences (including sociocultural factors), and (c) temperament/personality traits [19]. Stress and prejudice, as environmental and sociocultural experiences, can contribute to the development of EMSs in sexual and gender minority adolescents [24]. Experiences of discrimination and social rejection, both in family and school environments (distal stressors), lead to feelings of defectiveness/shame (proximal stressors), while chronic stress resulting from minority stress and daily microaggressions amplifies vulnerabilities and insecurities [18,33]. An SGM adolescent raised in a sociocultural context of oppression may perceive their own gender identity or sexual orientation as wrong [23]. This perception, shaped by cultural factors (e.g., TV shows, social rules, laws that restrict rights for SGM individuals), can contribute to the development of EMSs (e.g., as shown in Table 2). The rejection experienced in this context, along with violence, bullying, and discriminatory behaviors, may lead them to form negative beliefs about themselves and their identity. Furthermore, internalized homophobia and the lack of positive role models reinforce feelings of inadequacy and lower self-esteem [34]. These factors interact, increasing vulnerability to the development of EMSs.

## 3. Early Maladaptive Schemas and Unmet Emotional Needs in SGM Adolescents 

ST views EMSs as frameworks for interpreting reality, offering a stable and coherent sense of oneself and the world. Many of these schemas develop early in life and become more complex over time due to the natural tendency to interpret information in alignment with their content [19]. EMSs stem from traumas, abuse, and repeated negative experiences that hinder the fulfillment of basic emotional needs, including (a) secure attachments, (b) emotional validation, (c) autonomy, (d) realistic limits and self-control, and (e) spontaneity [19].

SGM individuals often struggle to have their emotional needs met, especially when lacking familial support that demonstrates affirmative and reparative attitudes [18]. Children and adolescents, particularly those in the SGM community, may face various adversities, including neglect and violence, especially from family members and close acquaintances.

Frequent negative experiences related to sexual orientation or gender identity during adolescence hinder the meeting of basic emotional needs (as shown in Table 1). The interplay of these experiences with emotional temperament leads to EMSs. For example, while heterosexual adolescents typically receive social and familial support, including their romantic relationships, SGM adolescents may conceal their identity to avoid backlash from an oppressive sociocultural context [33]

LGBTQIA+ adolescents may adopt strategies to fit in, such as participating in cis-heteronormative activities (e.g., dating the “oppositive sex”) or engage in hyper-masculine/feminine activities to hide their gender identity. These experiences, when not addressed affirmatively, can reinforce EMSs related to self-image and identity. Common beliefs among SGM adolescents facing an oppressive environment with invalidating sociocultural rules are illustrated in Table 2.

Schemas developed during childhood and adolescence remain active throughout life, particularly when an experience is unconsciously perceived as reminiscent of past traumatic experiences. In such instances, ST for Children and Adolescents (ST-CA) plays a crucial preventive role in relation to EMSs [35], as psychological issues during these developmental stages often foreshadow severe mental disorders in adulthood [36].

## 4. ST Affirmative Strategies for SGM Adolescents

The literature on interventions in ST for SGM individuals, including in ST-CA, remains scarce. A recent study addressing this topic by Cardoso and collaborators [18] presents characteristics common to the SGM population, such as the inner critic/oppressive sociocultural schema mode, and proposes strategies based on ST for adults belonging to the SGM population. In addition to this work, the literature on ST does not contain other studies that focus on working with SGM adolescents, which reaffirms the need to produce research with a focus on this approach. In ST-CA, working with schema modes and limited reparenting are highlighted as tools for expressing emotional needs and fostering an affirmative space [37]. Alongside mode work, other techniques can be adapted and applied to the SGM population [18]. It is crucial for therapists to understand the specific stressors and processes involved with SGM individuals [38], as well as to consider developmental particularities and the necessity of adapting session structures and therapeutic materials for adolescents [39]. Fundamental principles of affirmative practice must acknowledge that the sexual orientation and gender identity of LGBTQIA+ adolescents are not psychopathological processes. Additionally, any attempts to ‘cure’ these aspects of identity are both unethical and invalidating.

In this section, we will describe five affirmative technical strategies in ST-CA: (a) working with modes, (b) imagery rescripting, (c) chair work, (d) photo techniques, and (e) creative resources.

### 4.1. Working with Schema Modes

Working with schema modes has shown positive results by facilitating clients’ understanding of their functioning without needing to understand many EMSs and the feelings and behaviors resulting from the activation of each schema [40]. When working with children and adolescents, the language of schema modes is the most used and recommended because (a) children and adolescents can more easily identify their emotions and behaviors, (b) affective states can change more rapidly in children and adolescents, and (c) it is understood that clients’ EMSs in this age group are still forming, which can make intervention focusing on schemas impractical. Therefore, identifying maladaptive patterns through work with schema modes allows for the development of interventions in a more suitable and effective manner [35].

### 4.2. Imagery Rescripting

Imagery techniques play a crucial role in ST, especially when directed towards adolescent audiences. The primary purpose of these techniques is to facilitate an emotionally reparative experience regarding significant events in the development of EMSs [37]. When applied to adolescents belonging to SGMs, imagery techniques can be used to explore and assign new meanings to memories related to invalidation, lack of acceptance, and prejudice in general. During the execution of these techniques, it is essential for the therapist to adopt a reparenting approach, meeting the emotional needs of the client and helping them build new interpretations and meanings regarding stressful events [18,19].

For example, when addressing the vulnerable child mode, the client can share a childhood experience in which they felt deeply defective, abandoned, and helpless when discovering their sexual orientation. The therapist can explore this experience by guiding the client to visualize the childhood scene in detail, encouraging them to connect with their emotions, physiological sensations, and thoughts at that moment [19]. As the visualization progresses, the therapist identifies the client’s unmet emotional needs, such as the need for safety, validation, love, and emotional support. The therapist then intervenes in the scene, taking on the role of a protective, caring, and affirmative parental or caregiver figure: comforting, offering words of encouragement and validation, reassuring the client that they are not alone in being a queer teenager. Additionally, the therapist helps the client position themselves with their wise/competent mode, recognizing and strengthening their internal skills and resources to cope with challenging situations. The client is encouraged to visualize themselves as a resilient and strong person capable of overcoming the challenges they have faced. Throughout this technique, the therapist helps the client reconnect with their unmet emotional needs, offering limited reparenting and promoting a positive change in their defectiveness, abandonment, or emotional deprivation schema [18,37].

### 4.3. Chair Work

Another significant tool in ST is chair work, also known as mode dialogs. This technique aims to activate schema modes and is conducted with two or more chairs positioned in the room, where each chair represents a relevant schema mode for the case at hand [35]. The main goal is to strengthen the wise/competent mode, empowering it to meet the client’s basic emotional needs. It is important to note that the client may present inner critic modes, including the inner critic (sociocultural oppressive) schema mode, which is common in SGMs [18]. Through mode dialogs, it is possible to help the client to learn more about the roots, features, and meanings of the inner critic (sociocultural oppressive) messages, as the use of chairs serves as a marker for each mode [41,42]. In this sense, working with modes aims to provide strategies for the client to deal with these modes more effectively in their daily life, challenging and reducing the control of their critical modes and meeting the needs of the more vulnerable modes through the wise/competent mode, which becomes affirmative [35].

### 4.4. Photo Techniques

Incorporating photographs can be an effective strategy for clients who have difficulty visualizing mental images, serving as an experiential technique that not only stimulates the activation of schema modes related to significant moments in their development, but also facilitates limited reparenting and addresses basic emotional needs within the context depicted in the image. For example, considering an adolescent developing a schema of social isolation or defectiveness, a photo from their childhood can assist in applying limited reparenting to an event where they faced discrimination for being different from their peers or when they felt labeled as perverse and fundamentally defective, allowing for emotional redemption due to the creation of reparative emotional reprocessing with a new meaning for that experience [18,42].

### 4.5. Creative Resources to Work with SGM Adolescents

TV shows, music, manga, cartoons, games, movies, and other tools can serve a playful role when working with adolescents [42]. Engaging discussions with SGM adolescents about characters and themes depicted in these resources, such as an LGBTQIA+ protagonist in a movie or manga, can enhance the strengths of a wise and competent affirmative mode. The therapist can ask the adolescent the primary strengths of an affirmative, wise, and competent mode represented by the character, or what the adolescent can learn from the model portrayed in the illustration. The main idea is to work closely with the adolescent, taking into account their preferences, hobbies, and interests.

## 5. Conclusions

The purpose of this theoretical review was to explore intervention opportunities with adolescents belonging to SGMs from the perspective of ST and ST-CA. Furthermore, this review can help fill a gap in the theoretical framework of ST, providing a foundation for clinical practice and the development of new research focused on working with this population. Despite significant advances in understanding the sociocultural implications on negative mental health outcomes in adolescents belonging to SGM groups (mainly according to Affirmative Therapy), there is a shortage of studies evaluating ST constructs with minority social groups, including SGMs. SGM populations face several social adversities, violence, and prejudice; however, the current literature available on the application of ST and other therapeutic approaches tailored to their specific needs is still limited. The study by Cardoso et al. [18], which investigated the use of ST for SGMs, stands out. Nonetheless, there is a significant gap regarding the application of ST in adolescents of this population.

To address this need, future research should focus on exploring and identifying the most common demands and effective therapeutic techniques for this group, adopting an affirmative and non-harmful approach. It is crucial for this research to also consider the prevention of unintended aggressions and microaggressions by therapists in the therapy room, given the prejudice faced by this community. 

New research could also be conducted based on the reflections provided by this review. A research agenda could include the following: (a) longitudinal studies considering sociocultural aspects and their influences on the acquisition of EMSs in different ethnic and minority social groups; (b) randomized clinical trials with ST interventions for adolescents belonging to SGMs; (c) creating culturally sensitive assessment instruments for adolescents belonging to different minority social groups; (d) mediation studies to identify empirical relationships between minority stress, EMSs, mental health, and negative outcomes in SGMs; (e) evaluating the effects of bullying on the acquisition of EMSs and the maintenance of maladaptive strategies in SGMs; and (f) the development of assessments and interventions focusing on positive aspects of SGM adolescents’ health (e.g., early adaptive schemas, healthy adult strengths, quality of life) as a way to prevent negative health outcomes in this population. By directing efforts to understanding and meeting the specific emotional and psychological needs of SGM adolescents, ST-CA can play a fundamental role in supporting the fulfillment of basic emotional needs and the healthy development of these young individuals.

## Figures and Tables

**Table 1:** Basic emotional needs and negative experiences in SGM adolescents. Based on Cardoso et al. [18].

Basic Emotional Needs	Negative Experiences
Safe connections with others (safety, stability, care, and acceptance)	Unstable connections, refusal to accept gender identity and/or sexual orientation, unsafe environment, lack of social connections.
Freedom of speech, validation of emotions and needs	Hindered emotional expression, emotional restrictions, emotional invalidation.
Autonomy, competence, and sense of identity	Limited sense of self, fragile identity affirmation, and strengthening of feelings of being incompetent.
Spontaneity and play	Limited expressions of leisure and spontaneity. Social withdrawal, inhibition of behaviors and emotions associated with gender identity and sexual orientation.
Realistic limitations and self-control	Excessive emphasis on self-control to hide their gender identity and/or sexual orientation. Vulnerability to exploitation, confusion about personal rights, relationship challenges.
Self-coherence	Environments that go against affirmation of gender identity and/or sexual orientation, erasure of non-binary identity (viewing it as part of a trend), which may influence the development of a confused (or not coherent) view of the self, as if one were not part of the world/context.
Justice	Experiences that reinforce injustices, frequently based on systematic violence, no expectations of fair treatment.

**Table 2:** Early Maladaptive Schemas and common beliefs in SGM adolescents.

Early Maladaptive Schemas	Common Beliefs in SGM Adolescents
Abandonment	“My parents abandoned me because I am who I am, so I’ll be abandoned by other people as well”. “My relationships will never last, why bother trying”.
Emotional deprivation	“Nobody can understand or validate me and my feelings”.
Mistrust/abuse	“People will cheat or betray me on purpose because of who I am”.
Defectiveness/shame	“I’m a mistake, an anomaly”.
Social isolation	“I’m too different from the others and cannot fit in any group”.
Dependence/incompetence	“I am incompetent and incapable of achieving (heteronormative) adult life expectations”
Vulnerability to harm or illness	“As a queer teenager, I always need to be prepared for the worst. Things can always end badly for queers—illness, physical harm, homelessness, etc.”
Enmeshment/undeveloped Self	“I need to always be connected to my parents, even when they do not validate my sexual orientation or gender identity”.
Failure	“I’m less successful than people who aren’t LGBTQ+”.
Entitlement/grandiosity	“I am better than other gay men because I am not feminine”.
Insufficient self-control/self-discipline	“We’ve faced enough challenges; we deserve to indulge and enjoy without feeling guilty”. “Discipline is for straight/cis people, we deserve more fun”.
Subjugation	“It’s better not to live my sexuality/gender identity to avoid criticism or exclusion”. “I will avoid speaking out even when I experience violence/mistreatment, because others will retaliate or feel betrayed by me”.
Self-sacrifice	“It’s only right that I’m always there for others in the LGBTQ+ community”.
Approval-seeking/recognition-seeking	“I need my friends to approve of my sexuality/gender identity”. “I need constant approval and attention for how fabulous I am”.
Negativity/pessimism	“Things will never change, LGBTQ+ will never be accepted”.
Emotional inhibition	“It is wrong to show affection for someone of the same sex as mine”.
Unrelenting standards/hypercriticalness	“I need to do things perfectly, so as not to validate stereotypes about LGBTQ+ people”.
Punitiveness	“I deserve to be punished for being who I am”.

## Data Availability

No new data were created or analyzed in this study. Data sharing is not applicable to this article.

## References

[1] Kroeber A.L., Kluckhohn C. (1952). Culture: A Critical Review of Concepts and Definitions.

[2] Matsumoto D., Juang L. (2021). Culture and Psychology.

[3] Skinner B.F. (1971). Beyond Freedom and Dignity.

[4] Gopalkrishnan N., Babacan H. (2015). Cultural diversity and mental health. Australas. Psychiatry.

[5] Ryder A.G., Ban L.M., Chentsova-Dutton Y.E. (2011). Towards a Cultural–Clinical Psychology. Soc. Personal. Psychol. Compass.

[6] Pilkington P.D., Younan R., Karantzas G.C. (2023). Identifying the research priorities for schema therapy: A Delphi consensus study. Clin. Psychol. Psychother..

[7] Meyer I.H. (2016). Does an improved social environment for sexual and gender minorities have implications for a new minority stress research agenda?. Psychol. Sex. Rev..

[8] Feder S. Disclosure: Trans Lives on Screen [Film], Field of Vision; Bow and Arrow Entertainment 2020; Level Forward.

[9] Hunter J., Butler C., Cooper K. (2021). Gender minority stress in trans and gender diverse adolescents and young people. Clin. Child Psychol. Psychiatry.

[10] Fox K.R., Choukas-Bradley S., Salk R.H., Marshal M.P., Thoma B.C. (2020). Mental health among sexual and gender minority adolescents: Examining interactions with race and ethnicity. J. Consult. Clin. Psychol..

[11] Garofalo R. (2011). The Health of Lesbian, Gay, Bisexual, and Transgender People: Building a Foundation for Better Understanding.

[12] Di Giacomo E., Krausz M., Colmegna F., Aspesi F., Clerici M. (2018). Estimating the risk of attempted suicide among sexual minority youths: A systematic review and meta-analysis. JAMA Pediatr..

[13] Liu R.T., Sheehan A.E., Walsh R.F., Sanzari C.M., Cheek S.M., Hernandez E.M. (2019). Prevalence and correlates of non-suicidal self-injury among lesbian, gay, bisexual, and transgender individuals: A systematic review and meta-analysis. Clin. Psychol. Rev..

[14] Slater M.E., Godette D., Huang B., Ruan W.J., Kerridge B.T. (2017). Sexual orientation-based discrimination, excessive alcohol use, and substance use disorders among sexual minority adults. LGBT Health.

[15] Dyar C., Newcomb M.E., Mustanski B. (2019). Longitudinal associations between minority stressors and substance use among sexual and gender minority individuals. Drug Alcohol Depend..

[16] Kessler R.C., Angermeyer M., Anthony J.C., De Graaf R.O.N., Demyttenaere K., Gasquet I., Üstün T.B. (2007). Lifetime prevalence and age-of-onset distributions of mental disorders in the World Health Organization’s World Mental Health Survey Initiative. World Psychiatry.

[17] Bockting W.O., Miner M.H., Swinburne Romine R.E., Hamilton A., Coleman E. (2013). Stigma, mental health, and resilience in an online sample of the US transgender population. Am. J. Public Health.

[18] Cardoso B.L.A., Paim K., Catelan R.F., Liebross E.H. (2023). Minority stress and the inner critic/oppressive sociocultural schema mode among sexual and gender minorities. Curr. Psychol..

[19] Young J.E., Klosko J.S., Weishaar M.E. (2003). Schema Therapy: A Practitioner’s Guide.

[20] American Psychological Association (2022). Psychologist Workforce Capacity and Cultural Responsiveness. https://www.apa.org/workforce/publications/health-service-psychologists-survey/workforce-capacity-cultural-responsiveness.

[21] Psychology Board of Australia Registrant Data [June 2023]. Australian Health Practitioners Regulation Agency 2023. https://www.psychologyboard.gov.au/About/Statistics.aspx.

[22] Liu X., Yager I., Garcia Torres A. (2025). Queering Schema Therapy for Transgender and Gender Diverse Care. Gender Focused issue of the Australian Community Psychologist. Aust. Community Psychol..

[23] Meyer I.H. (1995). Minority stress and mental health in gay men. J. Health Soc. Behav..

[24] Meyer I.H. (2003). Prejudice, social stress, and mental health in lesbian, gay, and bisexual populations: Conceptual issues and research evidence. Psychol. Bull..

[25] Wong C.F., Schrager S.M., Holloway I.W., Meyer I.H., Kipke M.D. (2014). Minority stress experiences and psychological well-being: The impact of support from and connection to social networks within the Los Angeles house and ball communities. Prev. Sci..

[26] Fredriksen-Goldsen K.I., Emlet C.A., Kim H.J., Muraco A., Erosheva E.A., Goldsen J., Hoy-Ellis C.P. (2013). The physical and mental health of lesbian, gay male, and bisexual (LGB) older adults: The role of key health indicators and risk and protective factors. Gerontologist.

[27] Feinstein B.A., Wadsworth L.P., Davila J., Goldfried M.R. (2014). Do parental acceptance and family support moderate associations between dimensions of minority stress and depressive symptoms among lesbians and gay men?. Prof. Psychol. Res. Pract..

[28] Ryan C., Russell S.T., Huebner D., Diaz R., Sanchez J. (2010). Family acceptance in adolescence and the health of LGBT young adults. J. Child Adolesc. Psychiatr. Nurs..

[29] Santos J.J., Cerqueira-Santos E. (2020). Homofobia e Escola: Uma Revisão Sistematizada da Literatura. Rev. Subjetividades.

[30] Associação Brasileira de Lésbicas, Gays, Bissexuais, Travestis e Transexuais (2016). Pesquisa Nacional sobre o Ambiente Educacional no Brasil 2015: As Experiências de Adolescentes e Jovens Lésbicas, Gays, Bissexuais, Travestis e Transexuais em Nossos Ambientes Educacionais.

[31] Kosciw J.G., Clark C.M., Menard L. (2022). The 2021 National School Climate Survey: The Experiences of LGBTQ+ Youth in Our Nation’s Schools.

[32] Ruben A. (2018). LGBTQI Inclusive Education Report 2018.

[33] Katz-Wise S.L., Rosario M., Tsappis M. (2016). Lesbian, gay, bisexual, and transgender youth and family acceptance. Pediatr. Clin..

[34] Badenes-Ribera L., Fabris M.A., Longobardi C. (2018). The relationship between internalized homonegativity and body image concerns in sexual minority men: A meta-analysis. Psychol. Sex..

[35] Loose C., Graaf P., Zarbock G., Holt R.A. (2020). Schema Therapy for Children and Adolescents ST-CA: A Practitioner’s Guide.

[36] Wainer R., Wainer R., Paim K., Erdos R., Andriola R. (2016). O desenvolvimento da personalidade e suas tarefas evolutivas. Terapia Cognitiva Focada em Esquemas: Integração em Psicoterapia.

[37] Loose C., Zarbock G., Graaf P., Holt R., Loose C., Graaf P., Zarbock G., Holt R. (2020). Key theories and concepts in schema therapy and ST-CA. Schema Therapy for Children and Adolescents-ST-CA: A Practitioner’s Guide.

[38] Paveltchuck F., Catelan R., Catelan R., Sardinha S. (2023). Aplicações das terapias cognitivas e comportamentais no trabalho com minorias sexuais e de gênero. Manual de Gênero e Sexualidade na Psicoterapia: Fundamentos Teóricos e Intervenções Clínicas.

[39] Friedberg R.D., McClure J.M. (2015). Clinical Practice of Cognitive Therapy with Children and Adolescents: The Nuts and Bolts.

[40] Wainer R., Wainer G., Wainer R., Paim K., Erdos R., Andriola R. (2016). O trabalho com modos esquemáticos. Terapia Cognitiva Focada em Esquemas.

[41] Farrah J., Startup S., Heath G., Startup H. (2023). Creative use of mode dialogues with the Vulnerable Child and Dysfunctional Critical modes. Creative Methods in Schema Therapy: Advances and Innovation in Clinical Practice.

[42] Cardoso B.L.A., Campos J.G.F., Cardoso B.L.A., Paim K. (2023). Luca e o modo crítico (sociocultural opressor) internalizado: Contribuições da terapia do esquema para intervenção com minorias sociais. Terapia do Esquema no Cinema: Os Filmes e Séries na Compreensão da Prática Clínica.

